# *SlARF2a* plays a negative role in mediating axillary shoot formation

**DOI:** 10.1038/srep33728

**Published:** 2016-09-20

**Authors:** Tao Xu, Xin Liu, Rong Wang, Xiufen Dong, Xiaoxi Guan, Yanling Wang, Yun Jiang, Zihang Shi, Mingfang Qi, Tianlai Li

**Affiliations:** 1 College of Horticulture, Shenyang Agricultural University, Shenyang 110866, Liaoning, People’s Republic of China; 2 Key Laboratory of Protected Horticulture of Ministry of Education, No. 120 Dongling Road, Shenhe District 110866, People’s Republic of China; 3 Zunyi Normal University, No. 830 Shanghai Road, Zunyi City, Guizhou Province, People’s Republic of China.

## Abstract

*SlARF2a* is expressed in most plant organs, including roots, leaves, flowers and fruits. A detailed expression study revealed that *SlARF2a* is mainly expressed in the leaf nodes and cross-sections of the nodes indicated that *SlARF2a* expression is restricted to vascular organs. Decapitation or the application of 6-benzylaminopurine (BAP) can initially promote axillary shoots, during which *SlARF2a* expression is significantly reduced. Down-regulation of *SlARF2a* expression results in an increased frequency of dicotyledons and significantly increased lateral organ development. Stem anatomy studies have revealed significantly altered cambia and phloem in tomato plants expressing down-regulated levels of *ARF2a*, which is associated with obvious alterations in auxin distribution. Further analysis has revealed that altered auxin transport may occur via altered *pin* expression. To identify the interactions of AUX/IAA and TPL with ARF2a, four axillary shoot development repressors that are down-regulated during axillary shoot development, IAA3, IAA9, SlTPL1 and SlTPL6, were tested for their direct interactions with ARF2a. Although none of these repressors are directly involved in ARF2a activity, similar expression patterns of *IAA3*, *IAA9* and *ARF2a* implied they might work tightly in axillary shoot formation and other developmental processes.

Auxin, a simple phytohormone, is involved in numerous growth and developmental processes in plants. Specifically, indole-3-acetic acid (IAA) mediates apical dominance; stimulates the differentiation of vascular tissue; induces root initiation and lateral root development; mediates the tropistic responses; and exerts various effects on leaf and fruit abscission and fruit set, development, and ripening^1^,^2^,^3^. Molecular studies have revealed that auxin signaling is determined through the actions of three protein families: the TIR1/AFB auxin receptors^1^,^4^,^5^, AUXIN/INDOLE 3-ACETIC ACID (Aux/IAA) proteins and plant-specific transcription factor (B3-type) auxin response factors (ARFs). ARFs contain an N-terminal B3-derived DNA-binding domain (DBD), and the middle regions (MRs) determine their transcriptional activation or repression functions. A glutamine-rich MR acts as a transcriptional activator, whereas a proline and serine-rich MR acts as a transcriptional repressor^6^,^7^. The C-terminal domain (CTD) motifs III and IV of ARF are responsible for homodimerization or heterodimerization with other Aux/IAA proteins^8^. Under low auxin concentrations, the transcriptional function of ARF is repressed by direct interactions with Aux/IAA proteins^9^,^10^,^11^. When auxin concentrations are high, TIR1/AFB interacts with the Skp1–Cullin–F-box (SCF) E3 ubiquitin ligase^4^,^12^ to polyubiquitylate and target the Aux/IAA protein for degradation via the ubiquitin-mediated protein degradation pathway; subsequently, the repression on the ARF transcription factor is relieved and active auxin-dependent gene expression occurs^10^,^13^,^14^. However, some ARF transcriptional repressors do not interact or heterodimerize with Aux/IAA.

Recently, the transcriptional corepressors TOPLESS/TOPLESS-RELATED (TPL/TPR) have been shown to repress indeterminate meristem fates^15^ via interactions with different transcription factors, including AUX/IAA and ARF. BODENLOS (BDL) is an AUX/IAA protein that interacts with TPL to mediate root development. TPL cooperates with AUX/IAA proteins by binding the activating ARF to suppress the expression of auxin-responsive genes under low concentrations of auxin^16^,^17^. Moreover, AtARF2 and AtARF9, two transcriptional repressors, may also interact directly with TPL/TPR proteins, revealing that TPL/TPR co-repressors may occur as both TIR1/auxin-dependent and TIR1-independent, ARF-mediated repressors^18^.

Members of the *ARF* family (23 members in *Arabidopsis*) have been carefully examined. Given the extensive functional redundancy of ARF proteins, several single ARF mutations have quite profound altered phenotypes and developmental deficiencies^19^. *ARF3*, *ARF5* and *ARF7* T-DNA lines exhibit various auxin-related defects, including irregular gynoecium patterning, altered hypocotyl responses to blue light, and changes in auxin sensitivity, vascular development, and early embryogenesis^20^,^21^,^22^,^23^. *ARF2* and *ARF8* act as linkers between the ethylene and auxin signaling pathways, which regulate hypocotyl bending and mediate auxin homeostasis in hypocotyl elongation, respectively^24^,^25^,^26^,^27^. The *arf7 arf19* double mutant shows a more visible auxin-related phenotype not observed in *arf7* or *arf19* single mutants, with abolished lateral root development and hypocotyl gravitropism^19^,^28^.

In tomato, 22 putative functional *ARF* genes have been identified^29^. *ARF7* and *ARF9* affect fruit development by mediating cell division^30^,^31^. The reduced expression of *Sl-ARF4* improves the post-harvest behavior of tomato fruits by controlling sugar metabolism^32^; the overexpression of miR167 silences *ARF6* and *8*, resulting in female sterility^33^; and the down-regulation of *ARF2a* and *ARF2b* mediates fruit maturation^34^. Although these *ARF*s have been well characterized, the functions of other tomato *ARF*s remain unclear. Therefore, to understand the roles of ARF signaling networks in tomato development, increased knowledge of the function of other individual *ARFs* is needed.

Aerial organs, which originate from the shoot apical meristem (SAM), consist of three parts: an internode, a leaf, and an axillary meristem (AM) formed in the leaf axil^35^,^36^. The transport and distribute of auxin in the epidermal layer (L1 layer) of the SAM results in an auxin maxima, which induces leaf initiation, whereas an auxin minimum is required for axillary meristem formation^37^. High polar auxin transport promotes cell proliferation over differentiation and, thereby, meristem growth. Altering auxin distribution or auxin polar transport using an auxin transport inhibitor or an auxin transport/signaling mutant decreases the SAM size or inhibits the initiation of AMs and thereby interferes with shoot and inflorescence architecture^36^,^38^,^39^. The characterization of several tomato mutants defective in SAM and AM development revealed several transcription factors that are involved in AM initiation. The initiation of shoots and inflorescence by the lateral meristem is inhibited in tomato *blind (Bl*) mutants (*Bl* encodes an R2R3 MYB gene)^40^. Lateral suppressor (*Ls*) is expressed in leaflet axils, and *Ls* mutants completely lose their AM initiation capacity^41^. Another AM mutation in tomato is the *Goblet (Gob*) gene, a homolog to *CUP-SHAPED COTYLEDON1 (CUC1)/ CUC2* in *Arabidopsis thaliana*; the mutation of this gene results in the complete failure to initiate the vegetative AM and the down-regulation of *KNOX* gene transcription^42^,^43^. Further studies revealed that *Gob* is functionally parallel to *Bl* for axillary meristem initiation. In addition, *Lateral Suppressor*, *Blind* and *GOB* potentially mediate axillary shoot formation through immediate auxin distribution and signaling^44^,^45^,^46^.

PIN family proteins are known to be responsible for polar auxin transport. These proteins determine the fine auxin gradients established across the special organs for proper development. The Auxin transport mutant pin fails to form lateral organs as local auxin disorders accumulate. Auxin gradients are achieved through auxin polar transport, which instruct organ development in combination with auxin signal elements such as AUX/IAA and auxin response factor. Auxin transport is also subsequently affected by auxin response factor, which mediates PIN transcription levels. Although a single *nph4/arf 7* mutant showed no effect on auxin-induced PIN relocation, the *arf7 arf16 arf19* and *arf7 arf17 arf19* triple mutants exhibit significantly reduced auxin-dependent PIN relocation. The expression of PIN proteins is also reported to be mediated by auxin through the TIR1-Aux/IAA-ARF pathway. Polar auxin transport (PAT) and auxin responses are tightly interlinked and therefore difficult to resolve in plants^47^.

Precise auxin action is fine-tuned through these complex pathways. Auxin plays a vital role in AM formation, and the down-regulation of several auxin signals in plants, such as *IAA3* and *9*, *pin3* and *pin4*, can cause strongly modified phenotypes in axillary shoot formation^48^,^49^,^50^. However, little information is available about the special auxin transcriptional factor involved in this process.

Here, we used *P*_*SlARF2a*_::GUS and qRT-PCR to show that *SlARF2a* exhibits a wide range of expression during tomato development. *SlARF2a* is expressed in roots, leaves, flowers, fruits and seeds, which implies that it might be involved in major organ development in tomatoes. Moreover, *SlARF2a* expression is reduced during decapitation, and BAP treatment induces axillary shoot formation. The down-regulation of *SlARF2a* expression further supported the finding *SlARF2a* plays a negative role in axillary shoot meristem formation. Moreover, the increased frequency of polycotyledons and organ fusion, two auxin-related defects, were also observed in the *SlARF2a*RNAi lines. The alteration of auxin distribution and *pin* expression in *SlARF2a*RNAi lines may underlie these phenotypes. Finally, the relationships between *ARF2a*, *IAA3* and *IAA9* are discussed.

## Results

The *SlARF2a* (Solyc03g118290.2.1) gene contains a 2511-bp open reading frame that deduces an 846-amino acids protein. The SlARF2a protein contains three conserved domains: B3 (135–237), ARF (263–345) and Aux/IAA (709–803). SlARF2a is predicted to function as a transcriptional repressor, as regions with high percentages of proline (7.87%), serine (12.55%), and threonine (6.38%) were identified in the MR domain sequences (Supplementary Fig. S1).

**Figure 1:**
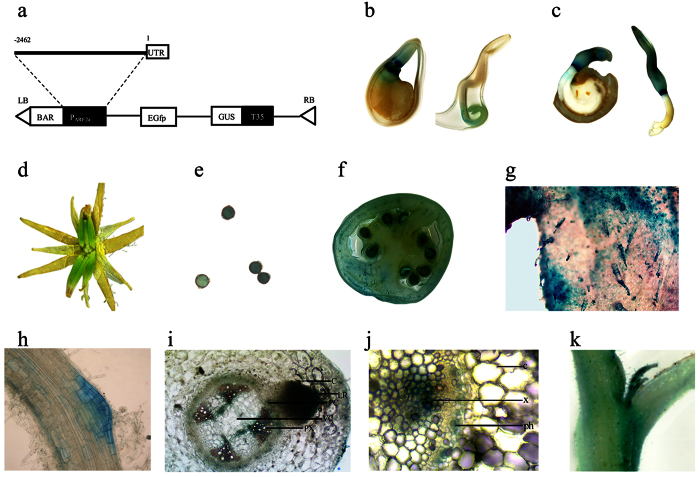
Tissue-specific expression of *SlARF2a* assessed in transgenic tomatoes expressing a GUS reporter gene driven by the *SlARF2a* promoter (P_*SlARF2a*_*::GUS*). The structure of the P_*SlARF2a*_*::GUS* vector (**a**); GUS staining patterns in germinated P_*SlARF2a*_*::GUS* seeds (2 days old) (**b**); GUS staining patterns in germinated DR5::GUS seeds (2 days old) (**c**); GUS staining patterns in the flowers of P_*SlARF2a*_*::GUS* plants at anthesis (**d**); pollen showed strong blue staining (**e**); MG was expressed in fruits, and the staining was mainly distributed in the vascular tissues and seeds (**f**); major GUS staining was noted in the leaf trichomes (**g**); GUS staining in the initial lateral root site (**h**); P_*SlARF2a*_*::GUS* expression in the lateral root and vascular root network (**i**); P_*SlARF2a*_*::GUS* expression in the vascular stem network (**j**); P_*SlARF2a*_*::GUS* expression in the axillary shoot (**k**).

**Figure 2:**
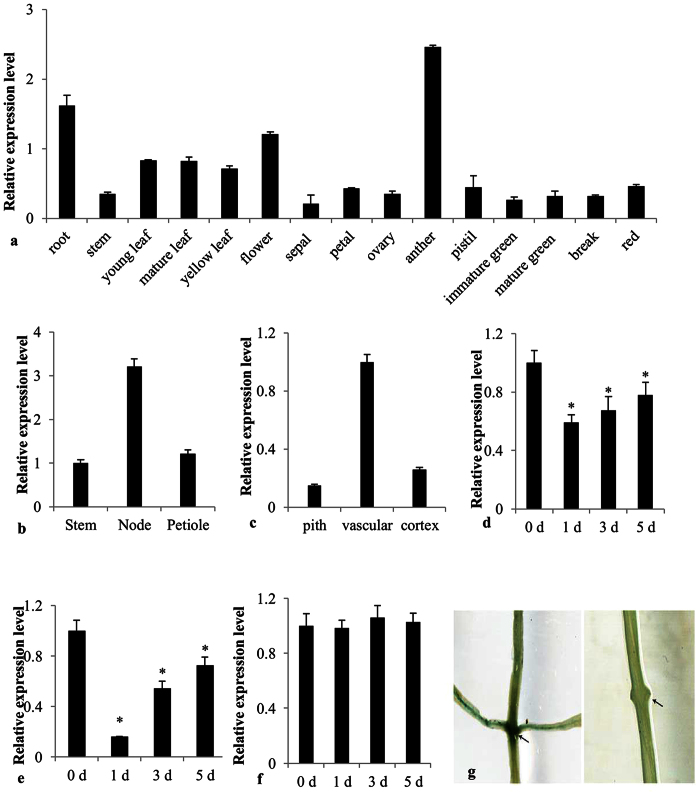
Analysis of *SlARF2a* expression patterns during tomato development. The different *SlARF2a* expression patterns in tomato organs (**a**); relative *SlARF2a* expression levels in stems, nodes and petioles of 6-week-old tomato plants (**b**); *SlARF2a* expression in the pith, vascular and cortex of stem nodes (**c**); *SlARF2a* expression 1, 3 and 5 days after decapitation (**d**); *SlARF2a* expression 1, 3 and 5 days after BAP treatment (**e**); *SlARF2a* expression 1, 3 and 5 days after decapitation and auxin treatment (**f**). Error bars indicate the means ± SE of at least three independent replicates, n ≥ 9. *Significant differences with P < 0.05 determined using a *t*-test. P_*ARF2a*_*::GUS* expression in the cotyledon (left) and after decapitation (right) (**g**).

To understand the functions of *SlARF2a* during tomato growth and development, we evaluated the *SlARF2a* expression patterns in various organs using GUS reporter gene fusion (Fig. 1a). *SlARF2a* is expressed in major plant organs, including seeds, roots, leaves, flowers and fruits. GUS activity was detected in 3-day-old, light-grown transgenic seedlings. During seed germination, the auxin reporter DR5 appeared in the radicle, whereas *SlARF2a*::*GUS* strictly appeared in the cotyledon (Fig. 1b,c). *SlARF2a*::*GUS* staining was detected in the stamen and stigma of the flower (Fig. 1d), and the pollen grain showed strong GUS activity (Fig. 1e). *SlARF2a:*:*GUS* was also expressed in the developing fruits, and GUS staining was mainly observed in the vascular tissues and seeds (Fig. 1f). *SlARF2a*::*GUS* was also strongly expressed in the leaf, but further analysis showed major staining in the trichome and strong expression in the root tip and lateral root formation sites (Fig. 1g,h). Root cross-sections revealed *ARF2a* expression in the vascular tissue and the epicycle (Fig. 1i,j). Staining was also observed in the branch (Fig. 1k). These expression profiles were confirmed by GUS activity and qRT-PCR analysis (Fig. 2a and Supplementary Figs S2 and S3). However, although low *SlARF2a* expression was detected in the stem, strong staining was detected in the leaf node, especially in the vascular tissue. The stem cross-section analyses indicated major *ARF2a* expression in vascular tissue (Fig. 2b,c).

During decapitation-induced axillary shoot development, *SlARF2a* showed a decreased expression trend. Auxin is transported basipetally down the shoot, and upon plant decapitation, the major source of auxin is removed. This removal of apical dominance stimulates axillary shoot formation within 4 d and *ARF2a* expression is significantly decreased during this process (Fig. 2d). The other axillary shoot was stimulated through BAP treatment on the axillary bud sites of the cotyledons, which effectively induced the axillary shoot development within 6 h. A pattern of down-regulated *ARF2a* expression was observed as 6-BA promoted axillary shoot development (Fig. 2e). The decrease in *ARF2a* expression was inhibited by the application of auxin on the cut surface (Fig. 2f). Moreover, the excision of immature leaves significantly stimulated axillary shoot development and decreased *ARF2a* expression (Fig. 2g).

**Figure 3:**
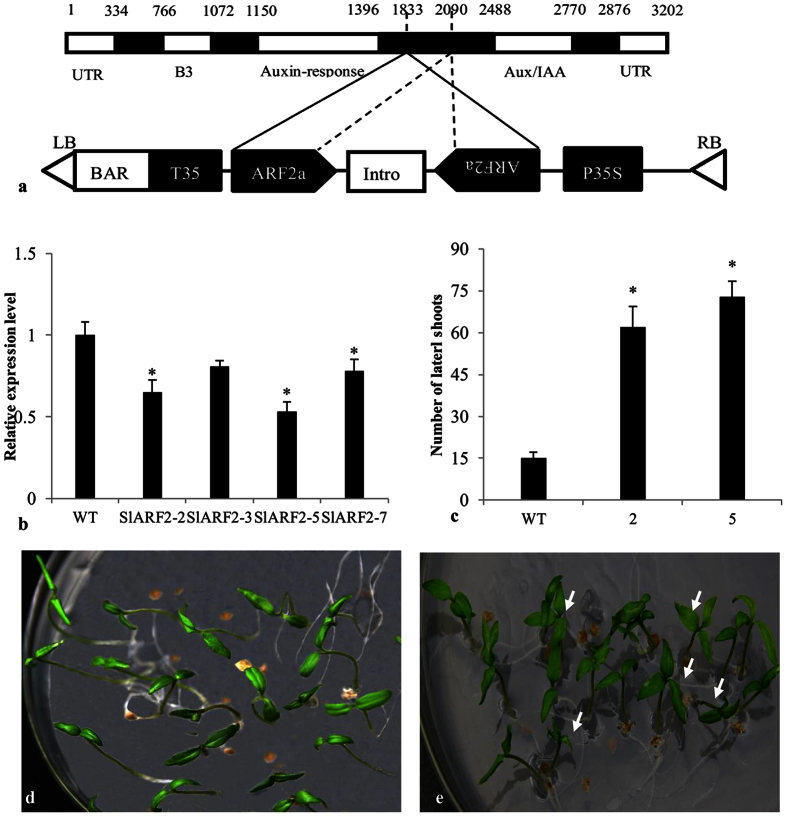
Down-regulation of *SlARF2a* alters cotyledon and axillary shoot development. The structure of the *SlARF2aRNAi* vector (**a**); relative *SlARF2a* expression levels in different *SlARF2aRNAi* lines (**b**); *SlARF2aRNAi-2* and *SlARF2aRNAi-5* promote the axillary shoot number (**c**); normal cotyledons in wild-type tomatoes (**d**); increased frequencies of triple and quadruple cotyledon phenotypes in SlARF2aRNAi lines (**e**). Error bars indicate the means ± SE. *Significant differences between transgenic and wild-type plants, with P < 0.05 determined using a *t*-test.

To further elucidate the function of *SlARF2a*, the transgenic *SlARF2aRNAi* lines were generated based on a 277-bp fragment (Fig. 3a). The fragment was cloned into an RNA binary vector (pB7GWIWG2(I)), which was then transferred into tomato using *Agrobacterium tumefaciens*. We obtained four independent RNAi lines in which ARF2a expression was down-regulated by more than 20% (e.g., RNAi*SlARF2a*-*2, 3, 5* and *7*). *SlARF2a*-*2* and *5*, which showed 38% and 42% reductions in *SlARF2a* transcript levels, respectively, were selected for further study (Fig. 3b).

*SlARF2aRNAi* down-regulation (lines 2 and 5) significantly promoted lateral branch development in all transgenic lines (Fig. 3c). Moreover, the *SlARF2a*RNAi lines had a higher frequency of polycotyledons than the wild-type plants. The polycotyledon frequencies were increased by 25% and 28% in *SlARF2aRNAi-2* and *SlARF2a*RNAi*-5* lines, respectively, compared with only 2% ectopic cotyledons in wild-type plants. Furthermore, approximately 15% and 17% of dicotyledons exhibit abnormal phenotypes in the RNAi*SlARF2a-2* and RNAi*SlARF2a-5* lines, respectively (Fig. 3d,e, Table 1).

**Figure 4:**
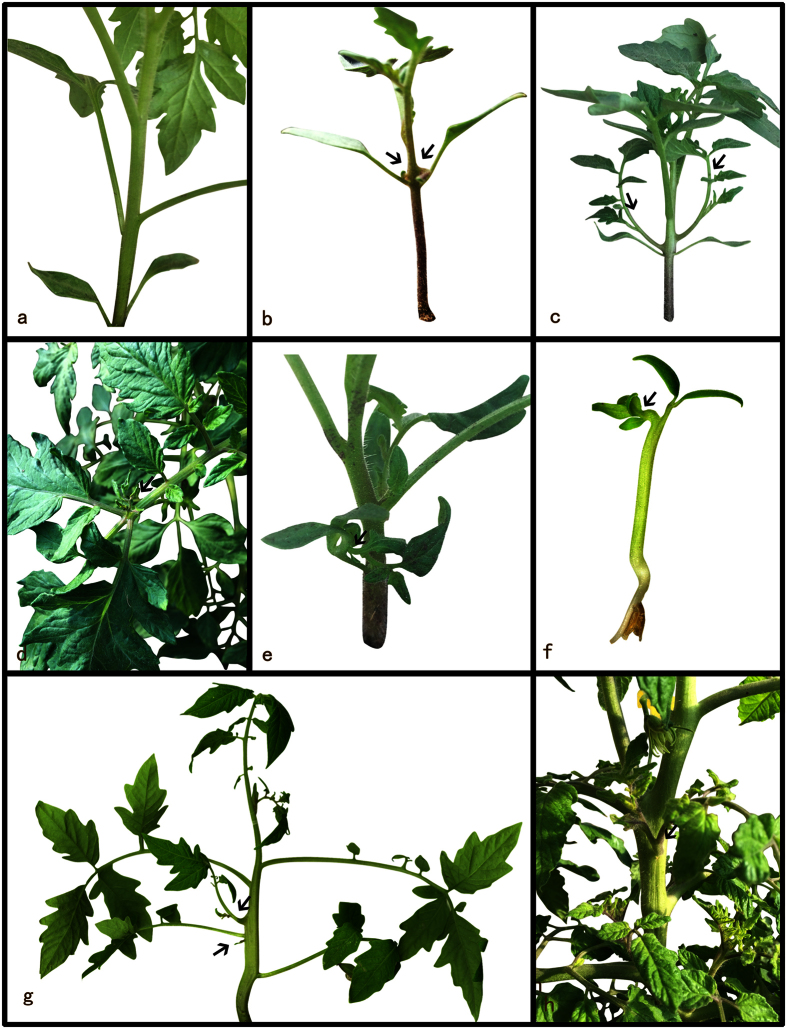
*SlARF2a* down-regulation promotes axillary shoot development. No axillary shoot formation in the cotyledon and leaf node before the first inflorescence appearance in wild type (**a**); *SlARF2aRNAi-5* plants showing the development of an unusual lateral branch inserted at a downward angle from the cotyledon node (**b**); the developed lateral branch in *SlARF2aRNAi-5* (**c**); *SlARF2a* down-regulation induced lateral branch formation at a site far from the leaf node (**d**); lateral branches originate on the abnormal twist and split cotyledons in a *SlARF2aRNAi-5* plant (**e**); two abnormal meristems in a *SlARF2aRNAi-5* seeding (**f**); unusual axillary shoot formation at a site far from the node in a *SlARF2aRNAi-5* plant (**g**); unusual axillary shoot formation in the stem of *SlARF2aRNAi-5* (**h**).

Normally, lateral shoots emergence occurs at the eighth leaf node only after the floral transition (Fig. 4a). In the transgenic *SlARF2aRNAi* line, lateral shoot emergence occurred at the first leaf node (Fig. 4b,c). An unusual meristem also appeared in the mature leaf (Fig. 4d). An exceptional phenomenon was observed in approximately 10% percent of *SlARF2aRNAi*-2 plants, in which lateral branches originated on the abnormal twist and split cotyledons but failed to mature due to cotyledon abscission (Fig. 4e). Moreover, in *SlARF2aRNAi*-2 lines with the greatest down-regulation, lateral shoot development appeared below the position of the cotyledon nodes (Fig. 4f). In addition, the unusual lateral branches also appeared in the stem, which is located far from the leaf node (Fig. 4g,h).

**Figure 5:**
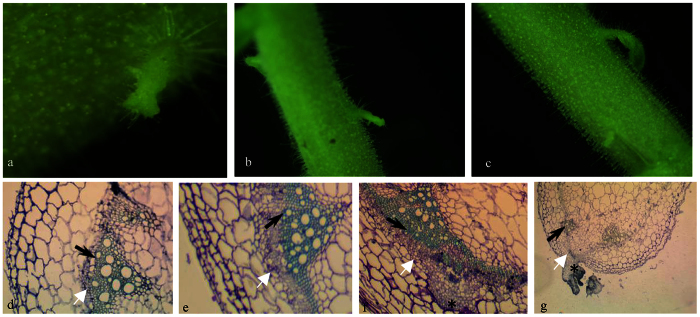
Abnormal axillary shoot meristems in *SlARF2aRNAi-5*. Enlarged view of a meristem-like organ on the stem surface (**a**); abnormal meristem-like structures appeared in the stem but at a site far from the node in *SlARF2aRNAi-5* (**b**); lateral branches break the stem surface in *SlARF2aRNAi-5* (**c**); anatomical analysis of wild-type stems (**d**); expanded phloem and cambium in *SlARF2aRNAi* (**e**); cross-section of pin-like structures revealing that the structures originate from the cambium in *SlARF2aRNAi* (**f**); cross-section of a meristem-like structure in *SlARF2aRNAi* (**g**). The star represents an axillary shoot; black arrows represent xylem; white arrows represent phloem.

These lateral branches initially produced only a single leaf with no visible apical meristem (Fig. 5a,b). When the leaves were completely expanded, the shoot meristem became visible and produced complete lateral branches that were similar to those reported earlier in *dgt* mutants. The site at which the unusual meristem emerged should be noted, as the epiderm typically appeared to be split along the axis (Fig. 5c). The initial lateral branch meristem was confirmed by anatomical observation. An anatomical analysis of the shoot revealed that the unusual axillary meristems originated from the cambium. The major difference between the wild-type and *ARF2a*RNAi plants involves vascular changes (Fig. 5d,e). The typical anatomic structure revealed abnormally enlarged interfascicular cambia and phloem in the tomato plants with down-regulated *ARF2a* (Fig. 5f,g). These results suggest that *ARF2a* down-regulation stimulates lateral shoot development and alters vascular development. Moreover, *ARF2a*RNAi increases the frequency of shoot fusion (Supplementary Fig. S4).

**Figure 6:**
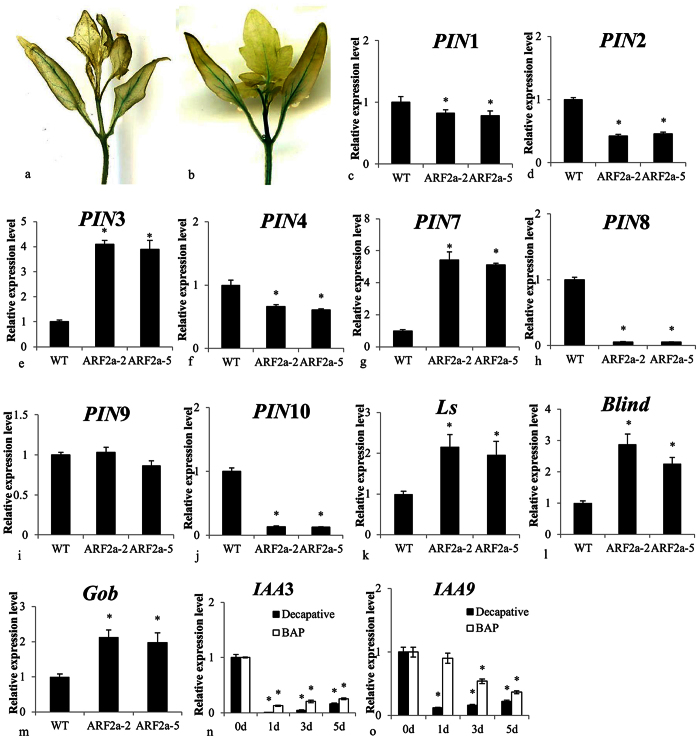
The distribution of auxin and the expression of *pin*, *blind*, *gob* and *ls* are altered in *SlARF2aRNAi* seedlings. *DR5::Gus* staining was observed in wild-type (**a**) and *SlARF2RNAi* (**b**). The *pin*1 (**c**), 2 (**d**), 3 (**e**), 4 (**f**), 7 (**g**), 8 (**h**), 9 (**i**), 10 (**j**), *blind* (**k**), *gob* (**l**) and *ls* (**m**) transcript levels in *SlARF2aRNAi* lines and wild-type were analyzed by qRT-PCR. Down-regulated IAA3 expression during decapitation and BAP-induced axillary shoot development (**n**); down-regulated IAA9 expression during decapitation and BAP-induced axillary shoot development (**o**). Error bars indicate the means ± SE of at least three independent replicates, n ≥ 9. *Significant differences between transgenic and wild-type plants, with P < 0.05 determined using a *t*-test.

Given that organ fusion and the formation of extra cotyledons and the axillary shoot are related to altered auxin signaling in specialized organs, the distribution of auxin was further examined in *ARF2a*RNAi lines via crosses with the *DR5::GUS* line, which is known as a good marker for studying auxin (Fig. 6a,b). An auxin transporter expression analysis indicated that *pins* expression was down-regulated in *ARF2aRNAi* (Fig. 6c–j). The results indicated that *pin1*, *2*, *4*, *8* and *10* were significantly down-regulated in *ARF2a*RNAi lines. In contrast, *pin9* expression was not altered, and *pin3* and *7* expression levels were up-regulated (*pin5* and *6* could not be detected). Several ectopic axillary shoot-related transcriptional factors have been identified in tomatoes, and *Blind*, *Gob and Ls* expression levels were significantly up-regulated in *ARF2aRNAi* (Fig. 6k–m). *IAA3* and *IAA9* expression levels were down-regulated after decapitation and BAP treatment (Fig. 6n,o). Similar expression trends for *SlTPL1* and *SlTPL6* were also noted in response to these treatments (Supplementary Fig. S5).

**Figure 7:**
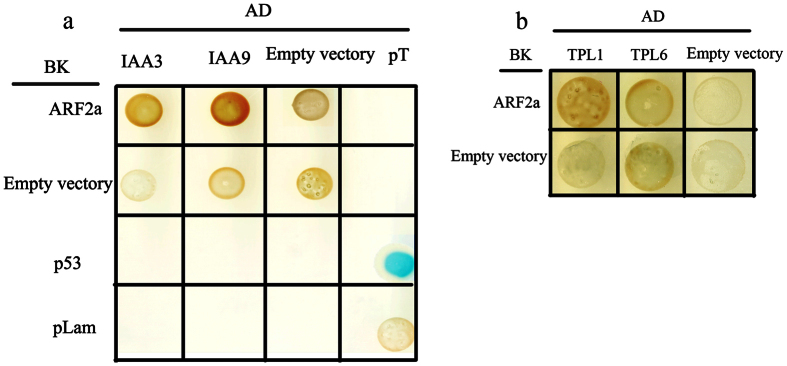
Yeast two-hybrid interactions between ARF2a and IAA3, IAA9, SlTPL1 and SlTPL6. The Y2HGold yeast strain was co-transformed with an ARF2a bait vector in combination with SlIAA3, SlIAA9, SlTPL1 and SlTPL6 prey vectors. Yeast two-hybrid interactions between ARF2a and IAA3 or IAA9 (**a**); yeast two-hybrid interactions between ARF2a and SlTPL1 or SlTPL6 (**b**).

The interactions between SlARF2a and SlIAA (3 and 9) and SlTPL (1 and 6) proteins were assessed using a yeast two-hybrid assay. We used full-length ARF2a, Aux/IAAs and SlTPLs to investigate the interaction. Blue colonies were obtained through plating onto QDO selective medium. Our results showed that ARF2a is incapable of interacting with SlTPLs (1, 6), SlIAA3, and SlIAA9 (Fig. 7).

## Discussion

Knowledge regarding the roles of ARF genes in plant morphogenesis have been obtained from their identification and characterization in *Arabidopsis.* Using T*-*DNA insertion tools, 22 *Arabidopsis ARFs* were obtained, and several mutants exhibited abnormal phenotypes^19^. For instance, the *arf3/ettin* mutant exhibits defects in floral development, while the *arf5/mp* mutant shows root meristem and cotyledon developmental defects^22^,^51^,^52^. The *arf7/nph4/msg1* and *arf19* mutants fail to undergo phototropic responses and lateral root development^19^,^23^,^24^,^53^. These results suggest functional redundancies among the ARF proteins. Compared with the *Arabidopsis* model plant, silencing a single ARF gene is sufficient to induce visible, stable and distinctive phenotypes in tomato*. Sl-ARF4*, *Sl-ARF7 and Sl-ARF9* are involved in fruit set, development, and quality, respectively^30^,^31^,^32^,^54^. The present study revealed that normal *ARF2a* expression is essential for axillary shoot and vascular development in tomato and thereby describes new roles for ARFs in tomato development.

Whole genome scanning identified two putative orthologs of *Arabidopsis ARF2* in the tomato genome. These proteins share high amino acid identity (83%). Silencing *SlARF2a* or *SlARF2b* separately resulted in fruit ripening defects, whereas the down-regulation of both genes led to severe ripening defects^34^. The proline-, serine- and threonine-rich regions in the MR domain sequences of *SlARF2a* are putative transcriptional repressors. The *DR5:*:GFP reporter experiment further supported these results, suggesting that SlARF2a might function as a transcriptional repressor in axillary shoot formation. The *SlARF2aRNAi* transgenic lines exhibited obvious phenotypes, thus further verifying the role of SlARF2 as a repressor. The report indicated that down-regulated *SlARF2a* expression is compensated for by the enhanced expression of *SlARF2b*, which exhibits discreet ethylene insensitivity during tomato fruit ripening. In this article, this compensation was not obvious in initial axillary shoot or cotyledon development. The low levels of *SlARF2b* expression in these organs might explain the lack of adequate compensation to restore deficiencies in ARF2a activity (Supplementary Fig. S6).

During seed germination, *ARF2a* is mainly expressed in the cotyledon, whereas auxin is mainly distributed in the radical. This distribution within the cotyledon implied that *ARF2a* is mainly involved in cotyledon development during the seedling stage. The *ARF2a*RNAi lines with down-regulated *ARF2a* exhibited an increased frequency of polycotyledon and axillary shoot formation. In our study, the most unusual finding involved the strong expression in the stem vascular tissue given the relatively low expression in the stem. Moreover, decapitation and the application of cytokinin, which promotes axillary shoot formation, significantly reduced *SlARF2a* expression. These expression patterns suggest that *ARF2a* might be involved in vascular development and axillary shoot formation. In addition, our results in tomato showed that *SlARF2a* is expressed especially in the fruits; notably, *SlARF2a* was recently reported to mediate tomato fruit ripening. These findings support the notion that *SlARF2a* is a major regulator of tomato development, which further implies that *ARF2a* might have a special and distinctive role in mediating tomato vegetable growth compared with other *ARF*s^30^,^31^,^32^,^34^,^54^.

The most obvious phenomenon in the *SlARF2a*RNAi lines was the significant increase in axillary shoots. The polar transport of auxin and the establishment of localized auxin maximal levels regulate embryonic development and shoot architecture. The major synthesized auxin originates from the young organ^55^,^56^ and is basipetally transported. Removal of the apical shoot leads to the depletion of original auxin levels and reduced auxin concentrations^36^,^57^,^58^,^59^. In *pin1*-null mutants, auxin gradients are not established, but can be restored by the application of auxin^37^,^60^,^61^. In *Arabidopsis thaliana* and tomato (*Solanum lycopersicum*), an auxin minima in the leaf axil is required for axillary meristem formation^36^. The application of auxin to the decapitation site interferes with PIN relocation, and polar auxin transport inhibits the increase in axillary shoots. The application of cytokinin to the leaf node effectively induces axillary shoot formation, as the low auxin concentration in the stem enhances cytokinin signals^36^,^62^,^63^. In the leaf node, *ARF2a* expression was especially decreased given that decapitation and cytokinin treatment induced axillary formation. These results imply that *ARF2a* might play a vital role in mediating axillary shoot formation. The down-regulation of *ARF2a* expression induces an abundant increase in axillary shoot formation even from the cotyledon nodes. Moreover, several lines exhibited abnormal ectopic axillary shoot formation in the cotyledon, leaf and stem, which further supported the notion that *ARF2a* plays a role in axillary shoot development. Moreover, organ emergence sites are regulated by the distribution of auxin, and controlled auxin redistribution is achieved through directional auxin transport^37^,^64^,^65^,^66^.

The changes in auxin distribution in *SlARF2a*RNAi plants are primarily attributed to the different auxin polar transport system in *SlARF2a*RNAi given that no significant difference in auxin content was noted in the seedlings (data not shown). Gene expression in response to auxin treatment occurs via the AUX/IAA-ARF pathway^57^,^67^. In axr3 mutants which Aux/IAA signaling is blocked, auxin could not induce PIN gene expression^47^. *IAA15* over-expression negatively regulates the abundance of auxin carriers at the transcriptional level, and perturbation of auxin homeostasis results in root gravitropism defects^68^. Moreover, two redundantly acting *ARF* transcription factors, *ARF5/MONOPTEROS (MP*) and *ARF7/NPH4*, jointly regulate both *pin1* expression and localization during lateral root patterning in *Arabidopsis*^22^,^23^. A *pin* expression analysis revealed that *pin1*, *2, 4, 8* and *10* are significantly down-regulated in *ARF2aRNAi*, whereas *pin3* and *7* expression is up-regulated. A previous report indicated that tomato *pin4RNAi*, the *dgt* mutant, and NPA (an auxin transport inhibitor that reduces auxin transport) induce greater axillary shoot development^50^,^69^. The strong down-regulation of *pin* expression might be a plausible explanation for the changes in the auxin gradient of *ARF2a*RNAi plants and the abundant axillary shoot development. Auxin accumulation is followed by the expression of the auxin transporter *pin*, which also mediates the first periclinal cell divisions and marks the onset of the interfascicular cambium^70^,^71^. Moreover, auxin signals are also involved in cambium initiation and activity^72^,^73^. *pin1* and *pin3* loss-of-function and auxin-insensitive *auxin resistant 1* mutants exhibit reduced or impaired interfascicular cambium initiation and activity^73^,^74^. In this study, the reason for enlargements of the vascular and interfascicular cambia in *ARF2aRNAi* might be due to the altered auxin distribution and *ARF2a*-dependent auxin signaling.

*Ls, Gob* and *Bl* mutants also mediate auxin distribution. The apices, leaves and stems of *Ls* plants accumulated more auxin compared to wild-type plants, and similar results have been found in *Bl* mutants except the leaves. *Gob* is also reported to possibly mediate auxin distribution, and its overexpression phenotype is affected by auxin-mediated *Gob* activity, revealing that auxin and *Gob* cooperation mediates leaf patterning^42^,^65^,^75^. The auxin signal is also involved in mediating their expression. The *SlIAA15* play a negative regulatory role upstream of *blind.* In seedlings with down-regulated *SlARF2a*, these key initial axillary shoot regulators exhibited increased expression, which potentially causes different auxin distribution patterns in *SlARF2aRNAi* plants. Furthermore, the high expression of *Ls*, *Gob* and *Bl* potentially cooperates with auxin to boost axillary shoot formation.

Normal auxin function via the ARF-Aux/IAA signaling pathway is required for tomato development. ARF transcriptional activity is mediated by AUX/IAA, and two tomato single AUX/IAA down-regulated lines, *IAA3* and *IAA9*, exhibit phenotypes similar to those of *ARF2a*RNAi, such as increased polycotyledon frequency, altered vascular formation and increased axillary shoot development^48^,^49^,^76^. It is reasonable to deduce that *ARF2a* might function under those AUX/IAAs. An earlier study suggested that *SlIAA3* is a linker between the auxin and ethylene signals that leads to enhanced differential growth and exaggerated hook curvature. Moreover, during this process, *SlIAA3* and *SlHLS* may act in parallel pathways, in which *ARF2* acts as a downstream component. Accordingly, *ARF2a* was significantly down-regulated in the AS-*SlIAA3* lines. These results indicate that *ARF2a* might contribute to the down-regulation of the *SlIAA3* phenotype. In addition, the role of *ARF2a* in the ethylene response is now clear given that *ARF2a*RNAi delays tomato fruit maturation by decreasing ethylene production and signaling, which may contribute to the reduced ethylene responsiveness and altered phenotypes observed in AS-*SlIAA3*. In this study, the use of decapitation and exogenous cytokinin to induce axillary formation also reduced *SlIAA3* expression. Thus, the putative IAA3-ARF2a pathway also functions in axillary development.

Increases in the vascular network and axillary shoot formation were also observed in AS-*SlIAA9* leaves. These findings indicate that *SlIAA9* down-regulation results in increased vascular differentiation and that *SlIAA9* is a key mediator in the auxin-dependent regulation of vascular vein patterning and lateral shoot development^48^. However, whether the activity or expression levels of *ARF2a* under *SlIAA9* directly mediate these processes remains unknown. An earlier report indicates that auxin-induced fruit set is affected by GA through the simultaneous down-regulation of *ARF2a* and *SlIAA9*, a finding that also reveals the close relationship between *ARF2a* and *SlIAA9*^77^. The down-regulation of these proteins during axillary shoot development after decapitation and exogenous cytokinin treatment implied that *ARF2a* acts downstream of IAA9. ARF2a is a potential central mediator of AUX/IAAs (at least SlIAA3 and SlIAA9) for tomato axillary shoot development and the ethylene response. Another view suggests that a fine and precise mechanism mediates cooperative *SlIAA3* and *SlARF2a* expression (along with *SlIAA9* and *SlARF2a* expression) to promote a proper response to developmental and environmental signals. The similar *cis-acting* elements found in the *SlIAA3*, *SlIAA9* and *SlARF2a* promoters might explain their similar expression patterns (Supplementary Table S3).

The lack of interaction among SlIAA3, SlIAA9 and SlARF2a suggests that SlARF2a transcriptional repression is not directly mediated by SlIAA3 and SlIAA9. Other reports also indicate that compared to other ARFs, only a few SlIAAs (SlIAA26 and 29) interact with SlARF2a^78^. Combined with the earlier report that the transcriptional repressor ARF might act without AUX/IAA repression given that very weak or no interaction was observed between the repressor ARF and AUX/IAA, these results indicate that ARF2a potentially functions as a transcriptional repressor in the absence of AUX/IAA.

These results have provided a framework for TPL/TPR-dependent transcriptional repression that is also involved in AUX/IAA-ARF-dependent auxin signaling. The interaction between Aux/IAA and TPL/TPR proteins to abolish ARF activity and inhibit auxin-responsive expression genes in low auxin concentrations indicates that TPL plays an important role in Aux/IAA-inhibited ARF transcriptional activity^16^. Although most ARF activators can directly interact with most Aux/IAAs, ARF repressors show minimal interactions with Aux/IAAs^17^,^79^,^80^, implying that ARF repressors are less affected by AUX/IAA compared to ARF activators. Further study revealed that AtARF2 and AtARF9, two repressive ARF proteins, can interact directly with TPL/TPR proteins to form co-repressors in mediating the auxin response, providing a new mechanism for repression and indicating that TPL/TPR act as co-repressors in both forms of ARF-mediated repression^17^. In tomato, IAA3 and IAA9 interact with all TPLs^18^. SlTPL1 and SlTPL6, which exhibit significantly down-regulated expression during axillary shoot development, did not directly interact with ARF2a in this study. It is reasonable to deduce that after decapitation or BAP treatment, SlTPL1 and SlTPL6 exhibited significantly decreased expression. Thus, fewer SlTPLs cooperate in transcriptional repression, and the reduced number of SlTPLs combined with the low expression levels of IAA3 and IAA9 resulted in reduced AUX/IAA-mediated repression of auxin signals and the release of more ARF activators. Thus, the coordinated accumulation of low levels of ARF2a leads to the release of ARF2a repression, and its binding site is available to ARF activators to activate the expression of auxin-responsive genes. Given that *ARF2a* activity is less affected by AUX/IAA and TPL and that this distinct ARF function is very rare (as most ARF activities are repressed by AUX/IAA and TPL), this finding implied that ARF2a activity is only dependent on itself at the transcriptional and translational levels; moreover, ARF2a might play a more direct role in adjusting auxin signals. Given that ARF2a expression primarily occurs in response to phytohormones, such as ethylene, abscisic acid and auxin^34^,^81^ (Supplementary Fig. S7 and Table S3), it is reasonable to deduce that *ARF2a* might be integral to those signals that direct tomato development.

## Methods

### Plant materials

Tomato cultivars (*Solanum lycopersicum* L. cv Zhongshu No 6) or “*Pro*_*ARF*2a_::*GUS*” and “*ARF*2a*RNA*i” transgenic lines were grown in soil for 6 weeks in a greenhouse with natural light under a daytime temperature of 25 ± 3 °C and a nighttime temperature of 15 ± 3 °C.

### Pro_ARF2a_::GUS and ARF2aRNAi vector construction and tomato transformation

The partial *ARF*2*a* clone was amplified using the following pairs of primers: *ARF*2*a* partial fw (5′-CACCAGACCATTCCCAAGCCAGTG-3′) and *ARF*2a partial rev (5′-TTGGTCCGCAGAGGGTAAAC-3′). The sequence was fused into the pENTR D-TOPO plasmid (Invitrogen) and then transferred to the binary vector pB7GWIWG2(I) via LR recombination according to the manufacturer’s instructions (Invitrogen). A 2.4-kb *ARF2a* promoter fragment was obtained by PCR using the following primers: Pro_*ARF2a*_ fw (5′-GGGGACAAGTTTGTACAAAAAAGCAGGCTTCGAAGGAGATAGAACCGTAATCATAATATCACGTCACATCGG-3′) and Pro_*ARF2a*_ rev (5′-GGGGACCACTTTGTACAAGAAAGCTGGGTT CACAAAATAAAACTTCCTTCTCCAAA-3′). The 2462-bp PCR product was transferred into pDONR221 (Invitrogen) and fused into the pBGWFS7 binary vector, which harbors two reporter genes for GUS (beta-glucuronidase) and GFP (green fluorescent protein) and the marker gene for bar. After sequencing, the vector with the correct sequence was electroporated into EHA105 cells. The T1 and T2 lines were obtained for the expression of the target and bar genes by qPCR using the primer pairs listed in Supplementary Table S1.

### RNA extract and expression assay

An RNAprep pure plant total RNA extraction kit (Qiagen, Germany) was used to extract total RNA. The genomic DNA was removed using DNase I and quantitative real-time PCR (qRT-PCR) analysis was carried according to Jain methods^82^. In brief, 2 μg cDNA samples were used as templates and were mixed with 200 nM of each primer and the SYBR Green PCR Master Mix (Qiagen, Germany) for RT-PCR analysis in an ABI 7500 Fast Real-Time PCR system (PE Applied Biosystems). The melting curve analysis was used to verify the reaction specificity. At least three independent biological replicates of each sample in technological triplicate were subjected to qRT-PCR.

### Statistical analysis

P < 0.05 and P < 0.01 were considered statistically significant according to Duncan’s Multiple Range Test. The Statistical Analysis System (SAS, version 9.1) was used for the data analyses.

### GUS analysis

For GUS staining, samples from T3 plants were incubated in pH 7.0 50 mM sodium phosphate solution containing 0.4 mg·ml^−1^ 5-bromo-4-chloro-3-indolyl-b-D-glucuronicacid, 1 mM potassium ferricyanide, and 1 mM potassium ferrocyanide for 5 h at 37 °C or 24 h at 4 °C, followed by incubation in 95% ethanol for 2 h. Pictures were obtained with a digital camera using a Nikon Eclipse 80i and a Zeiss Axio Observe A1 microscope.

### Light microscopy

The excised stem samples were immediately fixed in an FAA solution, dehydrated using a graded ethanol series (50, 60, 70, 90, 95 and 100%) for 30 min at each concentration, and embedded in paraffin. Paraffin-embedded sections (18–25 μm thick) were cut Using a Leica RM2245 microtome to obtain 8–25 μm thick sections, which were then de-paraffinized using 100% Histoclear. After Safranin-O or Toluidine blue staining, the samples were examined under a light microscope (Nikon Eclipse 80i).

### Hormone treatments

Decapitation treatment was performed by excising the shoot tip below the oldest unexpanded leaf while the remaining five leaves were allowed to expand. For the BAP treatments, 0.5 mM BAP was applied around the stem immediately below the oldest unexpanded leaf. IAA treatment was performed by applying lanolin containing 3 mg g^−1^ IAA to the decapitated stump.

### Yeast two-hybrid assays

The Matchmaker GAL4 Two-Hybrid System 3 (Clontech) was used for the yeast two-hybrid assays. The full-length sequences of *SlIAA3* and *9*, *SlTPL1* and *6,* and *SlARF2a* were obtained by PCR amplification (Supplementary Table S2). *ARF2a* PCR products were used to generate pGBKT7-*ARF2a* t. *IAA3, IAA9, SlTPL1* and *SlTPL6* PCR products were used to generate pGADT7- *IAA3, IAA9, SlTPL1* and *SlTPL6,* respectively. All constructs were verified by sequencing. Different pairs of pGBKT7-ARF and pGADT7- *IAA3, IAA9, SlTPL1* and *SlTPL6* vectors were co-transformed into the Y2HGold strain and selected on SD/-Leu/-Trp medium. The interactions between ARF2a and IAA3, IAA9, SlTPL1 and SlTPL6 were assayed on SD/-Ade/-His/-Leu/-Trp selective medium using at least 10 independent colonies.

## Additional Information

**How to cite this article**: Xu, T. *et al.* *SlARF2a* plays a negative role in mediating axillary shoot formation. *Sci. Rep.* **6**, 33728; doi: 10.1038/srep33728 (2016).

## Supplementary Material

Supplementary Information

## Figures and Tables

**Table 1:** Polycotyledon phenotype of *ARF2aRNAi*.

	Polycotyledon frequency	Abnormal dicotyledon frequency
WT	2 ± 1%^a^	1 ± %^a^
*RNAi-SlARF2a-2*	25 ± 5%^b^	15 ± 4%^b^
*RNAi-SlARF2a-5*	28 ± 6%^b^	17 ± 4%^b^

The polycotyledon phenotype occurs at a higher frequency in *ARF2aRNAi* lines compared with the wild-type (WT). Error bars represent the means ± SE (standard error) of 50 plants. ^a, b^Significant differences between transgenic and WT plants, with P < 0.05 determined using a *t*-test.
