# Supplementary material for: SlARF2a plays a negative role in mediating axillary shoot formation
Source: Sci Rep. 2016 Sep 20;6:33728. doi: 10.1038/srep33728 (PMC5028752; doi:10.1038/srep33728)
Supplement: Supplementary Information [file srep33728-s1.pdf]

Supporting Information

***SlARF2a* plays a negative role in mediating axillary shoot formation**

Tao Xu, Xin Liu, Rong Wang, Xiufen Dong, Xiaoxi Guan, Yanling Wang, Yun Jiang, Zihang Shi, Mingfang Qi, Tianlai Li

The following Supporting Information is available for this article:

Fig. S1 Alignment of the predicted protein sequences of SlARF2a and AtARF2. The DNA-binding domain (DBD) is underlined with a dashed line. The auxin response factor domain is underlined with a thick dashed line, and the conserved domains III and IV are indicated by thick lines. Identical and similar amino acids are shaded in black and gray, respectively.

Fig. S2 GUS expression (a) and activity analysis (b) in different P_{ARF2a}::GUS lines. The samples were obtained from lines 1, 5, 9 and 11. Standard errors are indicated (n=12).

Fig. S3 Analyzed of GUS relative expression (a) and activity (b) in different organs of PARF2a::GUS lines. The samples were obtained from lines 9 and 11. Standard errors are indicated (n=10).

Fig. S4 *SlARF2a* down-regulation induces organ fusion. White arrows indicate the fusion site. (a) Leaf and stem fusion in *SlARF2a RNAi-5*. (b) Axillary and stem fusion in *SlARF2a RNAi-5*.

Fig. S5 SlTPL expression after decapitation and BAP treatment. Relative mRNA levels in plant leaf nodes after treatment for 0, 1, 3 and 5 days. Standard errors are indicated (n=9). *, significantly different from the control, $P < 0.05$.

Fig. S6 Relative SlARF2a and SlARF2b mRNA expression levels in the root, stem, leaf, cotyledon, flower and fruit. Standard errors are indicated (n=6).

Fig. S7 Altered auxin expression in ARF2a lines. Relative mRNA levels of SlARF2a in tomato leaves collected 6 and 24 h after auxin (IAA) treatment. Untreated leaves were used as a control.

Standard errors are indicated (n=6). *, significantly different from the control, P<0.05.

Table S1 Tomato ARF accession numbers and qRT-PCR primer sequences for the indicated genes

Table S2 Primer sequences used for yeast two-hybrid assays

Table S3 Distribution of cis-acting elements in SlIAA3, SlIAA9 and SlARF2a promoter regions (5.0 kb)

Fig. S1 Alignment of the predicted protein sequences of SlARF2a and AtARF2. The DNA-binding domain (DBD) is underlined with a dashed line. The auxin response factor domain is underlined with a thick dashed line, and the conserved domains III and IV are indicated by thick lines. Identical and similar amino acids are shaded in black and gray, respectively.

```
AtARF2     MASSEVSMKGNRGGDNFSSSGFSDPKETRNVSVAGEGQKSNSTRSAAAERALDPEAALYRELWHACAGPLVTVPRQDDRV    80
SlARF2a    ..................MAASEVSIQGYSEPSDGSRPVSETGRSSSGVGIVDADTALYTELWRSCAGPLVTVPREGELV    62

AtARF2     FYFPQGHIEQVEASTNQAAEQQMPLYDLPSKLLCRVINVDLKAEADTDEVYAQITLLPEANQDENAIEKEAPLPPPPRFQ   160
SlARF2a    YYFPQGHIEQVEASTNQVADQQMPLYNLPSKILCRVVNVLLKAEPDTDEVYAQVTLMPEPNQDENAVKKEPMRPPPPRFH   142

AtARF2     VHSFCKTLTASDTSTHGGFSVLRRHADECLPPLDMSRQPPTQELVAKDLHANEWRFRHIFRGQPRRHLLQSGWSVFVSSK   240
SlARF2a    VHSFCKTLTASDTSTHGGFSVLRRHADECLPQLDMSRQPPTQELVAKDLHGNEWRFRHIFRGQPRRHLLQSGWSVFVSSK   222

AtARF2     RLVAGDAFIFLRGENGELRVGVRRAMRQQGNVPSSVISSHSMHLGVLATAWHAISTGTMFTVYYKPRTSPSEFIVPFDQY   320
SlARF2a    RLVAGDAFIFLRGENGELRVGVRRAMRQQGNAPSSVISSHSMHLGVLATAWHAIQTKTMFTVYYKPRTSPAEFIVPYDHY   302

AtARF2     MESVKNNYSIGMRFKMRFEGEEAPEQRFTGTIVGIEESDPTRWPKSKWRSLKVRWDETSSIPRPDRVSPWKVEPALAPPA   400
SlARF2a    MESVKNNYSIGMRFKMRFEGEEAPEQRFTGTIVGIEDADPQRWLESKWRCLKVRWDENSSIPRPDRVSPWKIEPALSPPA   382

AtARF2     LSPVPMPRPKRPRSNIAPSSPDSSMLTREGTTKANMDPLPASGLSRVLQGQEYSTLRTKHTESVECDAPENSVVWQSSAD   480
SlARF2a    LNVPPVARPKRPRSSILPTSPDSSVLTREGSSRATADHSQASGFPRVLQGQELSTFRGGFAEINETDLSEKPMIWQTSVN   462

AtARF2     DDKVDVVSGSRRYGSENWMSSARHEPTYTDLLSGFGTNIDPSHGQRIPFYDHSSSPSMPAKRILSDSEGKFDYLANQWQM   560
SlARF2a    DEKNDIHSASKRYLPDKWLPLGRPESSLTDLLSGFGSSHGFCLPSADQAAFGARLVKQQTQDQEKDFSLLGKPWSLLSSG   542

AtARF2     IHSGLSLKLHESPKVPAATDASLQGRCNVKYSEYPVLNGLSTENAGGNWPIRPRALNYYEEVVNAQAQAQAREQVTKQPF   640
SlARF2a    LSLNLMDSGSKAPGIGGDTPYQMRGDARYSGYGEFSVLPGHRVANQQGSWIMPQPVSPYMQLSSHSREMMHKPSVVKQPE   622

AtARF2     TIQEETAKSREGNCRLFGIPLTNNMNGTDSTMSQRNNLNDAAGLTQIASPKVQDLSD......QSKGSKSTNDHREQGRP   714
SlARF2a    AVKPK.....EGNYKLFGIPLTSNVCTDAVMMRKSSLIDPASDMNIGIHPHQSLATDSDQRSEQSKGSKVDDGVAANDHD   697

AtARF2     FQTNNPHPKDAQTKTNSS....RSCTKVHKQGIALGRSVDLSKFQNYEELVAELDRLFEFNGELMAPKKDWLIVYTDEEN   790
SlARF2a    KQFHTFHLAARDKDGKGHSSSTRSCTKVHKQGTALGRSVDLAKFNNYDELIAELDQLFDFNGELKARSKSWLVVYTDDEG   777

AtARF2     DMMLVGDDPWQEFCCMVRKIFIYTKEEVRKMNPGTLSCRSEEEAVVGEGSDAKDAKSASNPSLSSAGN               858
SlARF2a    DMMLVGDDPWQEFCGMVRKIFIYTKEEVQRMNPGTLNSKGEDTSSVAEGSDAKEVKNLQLPSESGQAE               845
```

Fig. S2

GUS expression (a) and activity analysis (b) in different P_{ARF2a}::GUS lines. The samples were obtained from lines 1, 5, 9 and 11. Standard errors are indicated (n=12).

a

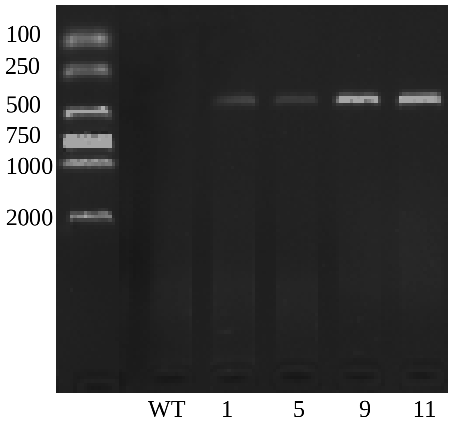

b

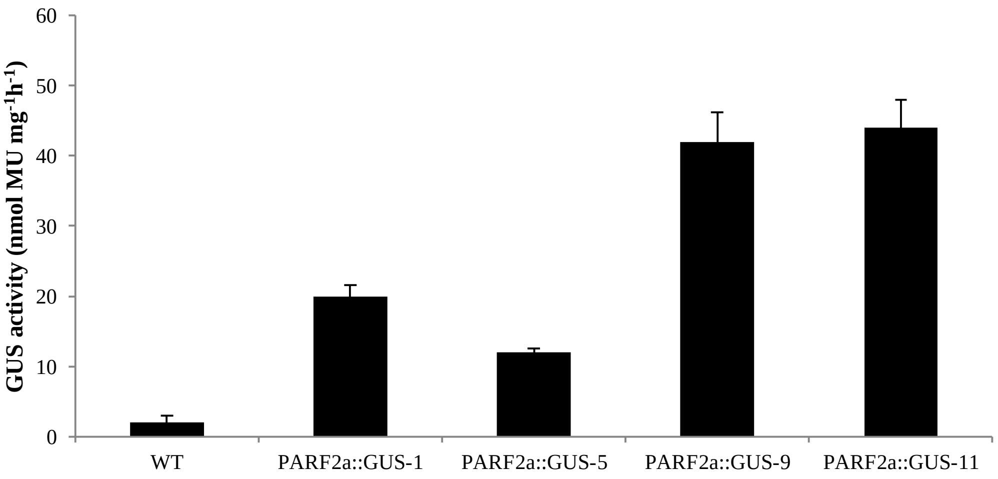

Fig. S3 Analyzed of GUS relative expression (a) and activity (b) in different organs of PARF2a::GUS lines. The samples were obtained from lines 9 and 11. Standard errors are indicated (n=10).

a

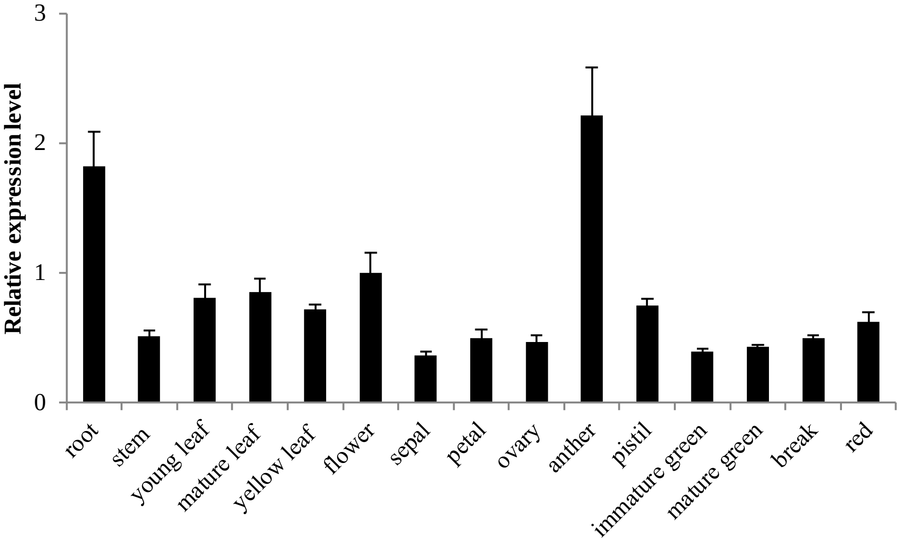

Relative expression level
3
2
1
0
root
stem
young leaf
mature leaf
yellow leaf
flower
sepal
petal
ovary
anther
pistil
immature green
mature green
break
red

b

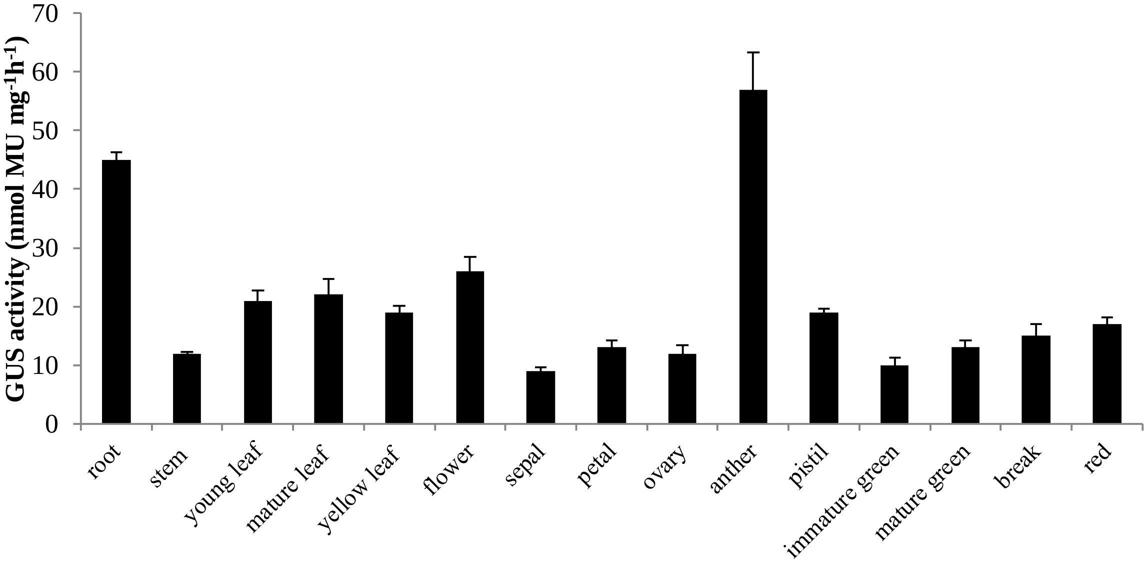

GUS activity (nmol MU mg-1h-1)
70
60
50
40
30
20
10
0
root
stem
young leaf
mature leaf
yellow leaf
flower
sepal
petal
ovary
anther
pistil
immature green
mature green
break
red

Fig. S4 *SlARF2a* down-regulation induces organ fusion. White arrows indicate the fusion site. (a) Leaf and stem fusion in *SlARF2a RNAi-5*. (b) Axillary and stem fusion in *SlARF2a RNAi-5*.

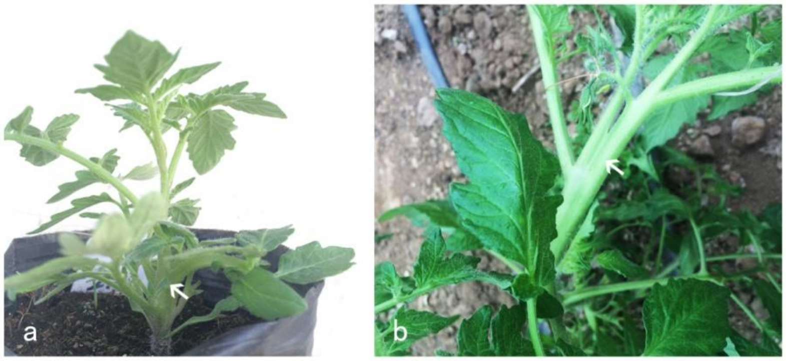

Fig. S5 SlTPL expression after decapitation and BAP treatment. Relative mRNA levels in plant leaf nodes after treatment for 0, 1, 3 and 5 days. Standard errors are indicated (n=9). *, significantly different from the control, P<0.05.

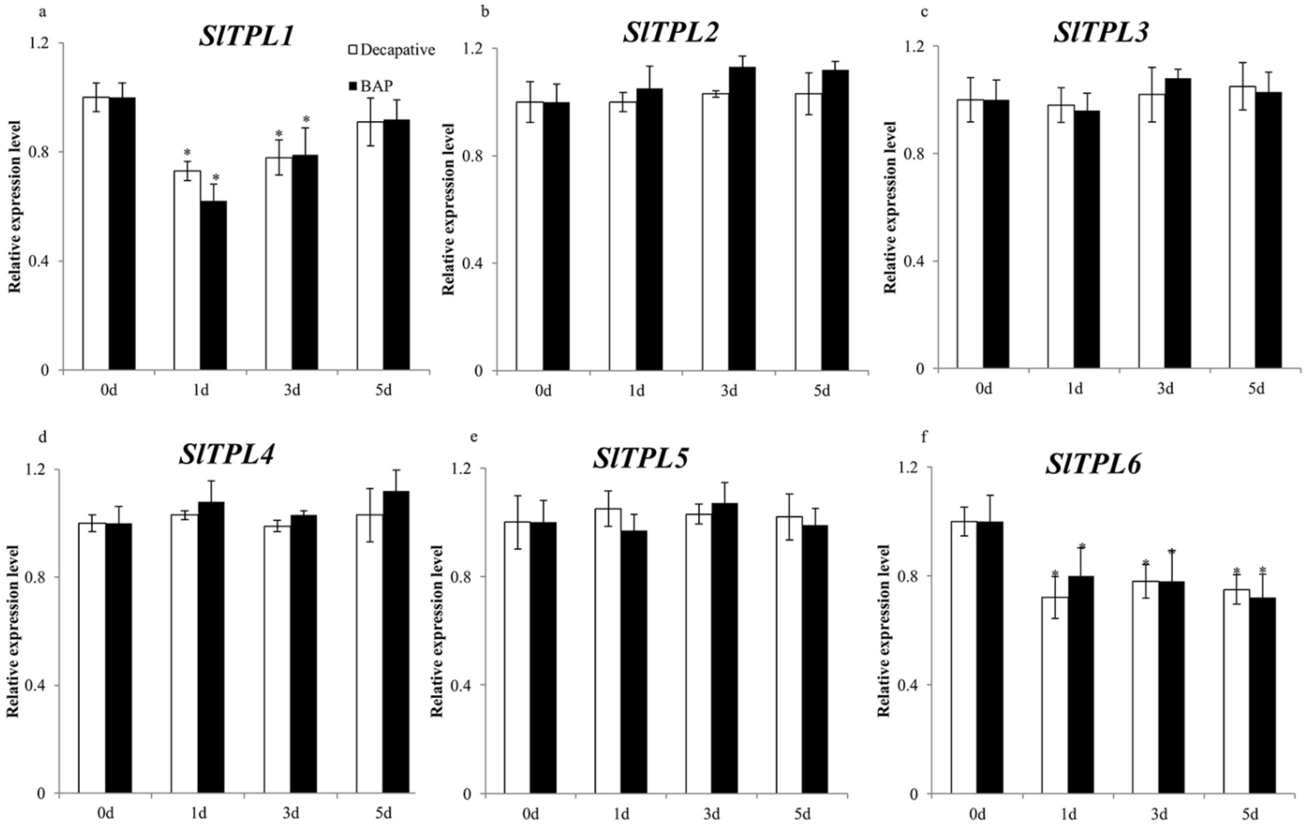

Fig. S6 Relative SlARF2a and SlARF2b mRNA expression levels in the root, stem, leaf, cotyledon, flower and fruit. Standard errors are indicated (n=6).

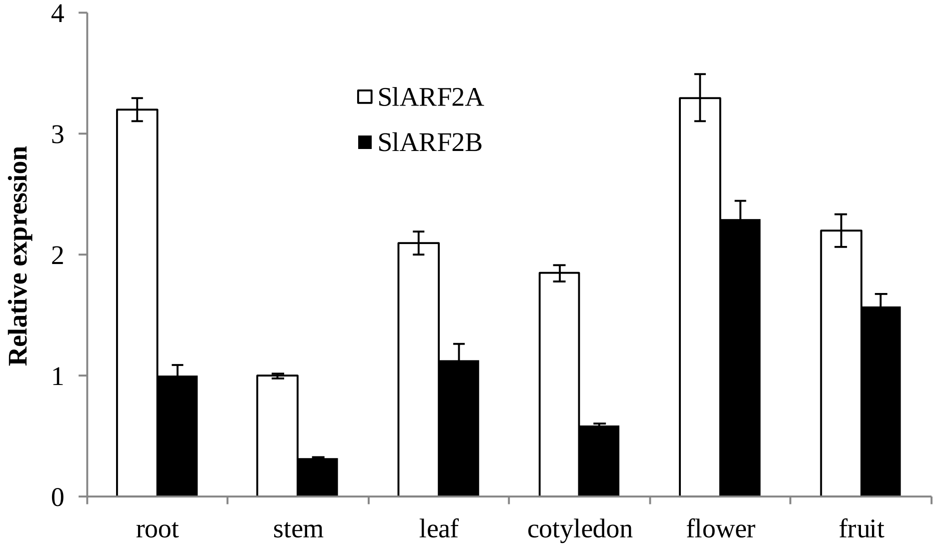

Fig. S7 Altered auxin expression in ARF2a lines. Relative mRNA levels of SlARF2a in tomato leaves collected 6 and 24 h after auxin (IAA) treatment. Untreated leaves were used as a control. Standard errors are indicated (n=6). *, significantly different from the control, P<0.05.

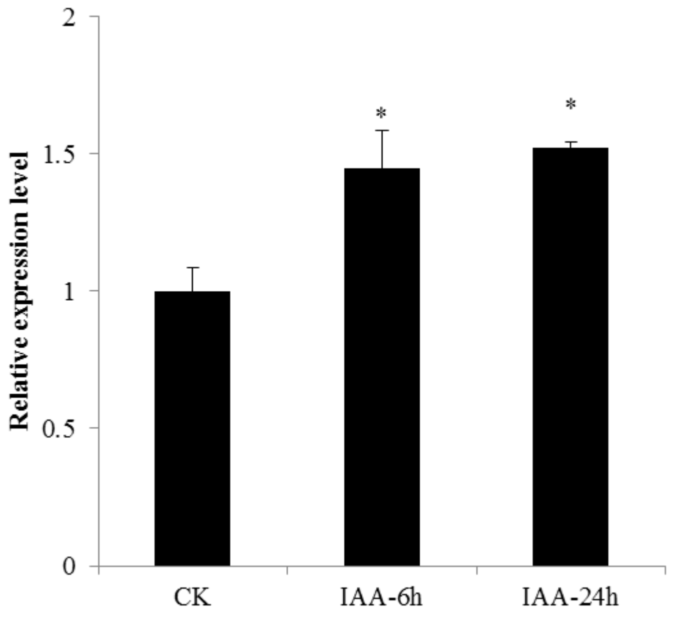

Table S1 Tomato ARF accession numbers and qRT-PCR primer sequences for the indicated genes

Gene name	Primer
*SlARF*2a F	GCAAGGTCAAGAGTTATCGA
*SlARF*2a R	CATTGGTTTCTGAGACAAGTC
*SlARF*2b F	TTTAACGAGTATCCAACCTTCC
*SlARF*2b R	GGGTTTAGGCATAATTTCTCCA
Actin F	CTTTGCGCATGCCATTCTT
Actin R	CGCAACCTCTCAGCACCAAT
SlTPL1 F	TGTTCGTTCTAGGAGACTAACCAG
SlTPL1 R	AAGACAAACCTTCCCTTCCGA
SlTPL2 F	CCTGTAAATACGCCTCTTGCT
SlTPL2 R	ACTGGTTGG AATGGACTGTG
SlTPL3 F	CACTTTCTGCTCCAATAACCT
SlTPL3 R	TCCA TCTGTCAACCCAACTG
SlTPL4 F	CCTTCTAACCCAAGCTCCAG
SlTPL4 R	ATAAACTCCGCCATCAGTAAGTC
SlTPL5 F	CGTCTATT GTAACCCATCCACTC
SlTPL5 R	AGAAGTTACACCATGAGGACCC
SlTPL6 F	ACTGGACTAGCATTCTCTAACAC
SlTPL6 R	TTGAATTCCACACCACTATCTGAG
Ls F	CCACCACCACCATCACTACC
Ls R	AGGGAAAGTGCGCGAGTAAA
Gob F	GTGGGGCCACCAATGGATTA
Gob R	TGGCTCAATCACCAAAGCCA
BLIND F	CGGCGCAATTACCTGGAAGA
BLIND R	GGCTGTTCTGGGCCTGTAAT
SlPIN1 F	TGGGTATATCAAGAACATAGCAAAG
SlPIN1 R	GCATTAAATATTAATTTGCATGGAA
SlPIN2 F	TTTTGCTTTTTACTCAAACACAGC
SlPIN2 R	TATTCCCCATGCCCCAAGT
SlPIN3 F	TTTCTCCCAAATCACAAAC
SlPIN3 R	GACACGCTAAATTGATTTTGAA
SlPIN4 F	GATTTGACAATTTTCTTCTCTTTT
SlPIN4 R	TCCTTTCTTCACTTGGCATA
SlPIN5 F	CCCCAAATTCACAACAAAAA
SlPIN5 R	CGATGAACACTTACTAGCGTCT
SlPIN6 F	TCAATCAACCCTTTCACTTTCTT
SlPIN6 R	GGGGGCCAAAGATTTTCTTA
SlPIN7 F	TCTGATATTCTAGAGAAAAAGAAGAAG
SlPIN7 R	TTTCCTTTTCAGATTACAGCTT
SlPIN8 F	TTTCGACATACAAAAATCTATAAGAAA
SlPIN8 R	TGCGATGACCATGTCTGAGT
SlPIN9 F	
SlPIN9 R	

SlPIN10 F	TGATCTGTACTTTGTTTTGTGAA
SlPIN10 R	GGTTCTGGCTCTTCATCATCA
	TTCATCACAACTAGCAACCAAA
	TTCATCACAACTAGCAACCAAA

Table S2 Primer sequences used for yeast two-hybrid assays

Gene name	Primer
ARF2a-F-ECORI	CCGGAATTCATGGCTGCTTCGGAGG
ARF2a-R-BAMHI	CGCGGATCCCTAAGATTCTGCTTGACCAGATTC
*IAA3*F-ECORI	CCGGAATTCATGAGAATTTACGAGAAGGATATCAAT
*IAA3*R-BAMHI	CGCGGATCCTTATAGACATGCTAGACCTTTAGCTTC
IAA9 F-ECORI	CCGGAATTCATGACAAGCGTGTTGGGTG
*IAA9*R-BAMHI	CGCGGATCCTTAACTCCTGTTCTTGCATTTCTC
*SLTPL1*F–ECORI	CCGGAATTCATGTCATCTCTCAGTAGAGAGCTT
*SLTPL1*R– BAMHI	CGCGGATCCTCATCTTGGTGCTTGATCGGAG
*SLTPL6*F-sfiI	CGCGGCCATGGAGGCCATGTCTCTTAGTAAGGACCTTAT
*SLTPL6*R-XmaI	CCCCCCGGGCTATATTGGTTGCTCATTGGTAA

Table S3 Distribution of cis-acting elements in SlIAA3, SlIAA9 and SlARF2a promoter regions (5.0 kb)

Table S3

Site name	Number of sites			Description and organism
	IAA3	IAA9	ARF2a	
ERE	3	1	3	*Dianthus caryophyllus* ethylene-responsive element
ABRE	3	3	1	*Arabidopsis thaliana* cis-acting element involved in abscisic acid responsiveness
GARE-motif		2	1	*Brassica oleracea* gibberellin-responsive element
TCA-element	1	1	2	*Nicotiana tabacum* cis-acting element involved in salicylic acid responsiveness
TGACG-motif	2	2	2	*Hordeum vulgare* cis-acting regulatory element involved in MeJA responsiveness
CGTCA-motif	1	1	1	*Hordeum vulgare* cis-acting regulatory element involved in MeJA responsiveness
3-AF1 binding site	1		1	*Solanum tuberosum* light-responsive element
ACE	3	1	1	*Petroselinum crispum* cis-acting element involved in light responsiveness
Box 4	3	4	4	*Part* of a conserved DNA module in *Petroselinum crispum* involved in light responsiveness
Box I	3	3	3	*Pisum sativum* light-responsive element
TCT-motif	1	1		Part of a light-responsive element in *Arabidopsis thaliana*
G-Box	1	3	2	*Antirrhinum majus* cis-acting regulatory element involved in light responsiveness
